# Initial clinical experiences of the muscle-preserving double door cervical laminoplasty with adjustable mini plates

**DOI:** 10.3389/fsurg.2022.1049937

**Published:** 2023-01-16

**Authors:** Wenliang Wu, Shuai Zhang, Tingbin Yan

**Affiliations:** Department of Orthopaedics, Qilu Hospital of Shandong University, Jinan, China

**Keywords:** cervical laminoplasty, cervical spondylotic myelopathy, minimal invasive surgery, muscle spaces, mini plates

## Abstract

Shirashi's double door laminoplasty method was a popular decompression procedure for cervical myelopathy. In this paper, we introduced a modified double door laminoplasty based on Shirashi's method with preliminary results. This study retrospectively analyzed 22 patients who underwent modified double door laminoplasty. During procedure, a single segment of the unilateral lamina was separated from the cervical semispinalis muscle and the multifidus muscle space for the preparation of lamina groove. A self-developed mini titanium plate was used to fix the inner side of the spinous process to complete the fixation after open-door process. The VAS, JOA scores and QoL scale were recorded for pain assessment, neurological and functional recovery. The overall curvature and range of motion of C2–C7 were measured with x-ray images. Changes in sagittal diameter of spinal canal were measured by CT scans. MRI was used to measure the cross-sectional area of cervical paravertebral muscles. All 22 patients successfully recovered with this procedure. The mean operation time, blood loss and follow-up durations were 117 ± 25 min, 149 ± 32 ml and 16.1 ± 3.6 months respectively. The preoperative, 3-month postoperative and 12-month postoperative JOA scores were 9.35 ± 3.25, 13.74 ± 4.86 and 15.73 ± 5.19 respectively. with improvement rates of 57.4% and 83.4%. Mean VAS scores before, 3 months after and 12 months after surgery were 1.81 ± 0.79, 2.82 ± 1.56 and 2.18 ± 1.34 respectively. The C2–7 lordotic angle and overall range of motion shows no statistical difference preoperatively and 12 post-surgery. The average sagittal diameter of the cervical spinal canal was enlarged from 9.15 ± 1.55 mm to 14.25 ± 1.46 mm. The average area of cervical paravertebral volume measured preoperatively and 3 months post operation was 84% of pre-operative value respectively. This value was improved to 93% of the preoperative value at 12 months post-surgery. This paper introduced initial experience on a modified posterior cervical double-door laminoplasty that was based on Shirashi's method, featuring creating bilateral laminar grooves on both sides and fixing central gap with self-developed mini plates. This procedure prevented obvious axial symptoms and improved patients' quality of life, which provided a baseline for further research with larger cohorts.

## Introduction

Posterior cervical laminoplasty is a common procedure for treatment of patients with extensive spinal cord compression (1). Today, there are two main types of procedures—the open-door laminoplasty proposed by Hirabayashi (2), and the double-door laminoplasty proposed by Kurokawa (3)—that are similar in terms of neurological recovery and postoperative range of motion. On the other hand, both procedures have a certain probability of postoperative axial symptoms, —persistent neck and shoulder girdle pain—which have a significant impact on patients' postoperative life (4–8).

In order to improve axial symptoms and better preserve the curvature of the cervical spine, Shirashi proposed a double door laminoplasty-based improvement that preserved deep extensor muscles, through exposing the central structure by splitting the spinous process—so as to avoid stripping all the muscle attachment from the spinous process—and exposing the lamina by separating the muscle space on both sides (7, 9). The postoperative cervical spine function and quality of life scores of these patients were significantly better than those of the traditional surgery group, throughout the minimum 2-year follow-up period. The better postoperative recovery of this new double-door laminoplasty was corroborated by subsequent surgical studies (4, 5). Furthermore, double-door laminoplasty has proven more advantageous than single-door laminoplasty (10). Yet in the Shirashi method, muscle is stripped on both sides after the spinous process is split, creating a certain degree of muscle atrophy after surgery.

The maintenance of opened lamina originated from suture fixation in the earlier reports of laminoplasty, which had a certain probability of lamina re-closure. To better maintain the decompression effect, spacers from autologous bone and hydroxyapatite (11), lateral mass suture anchors (12), as well as titanium plates (13) have been used in clinical practice with corresponding technical features. Most methods attained an effective long-term stabilization effect, yet the early firmness of internal fixation titanium plates was better than that of spacers fixed by suture.

In this paper, we improved the Shirashi double door laminoplasty method by preparing bilateral laminar grooves through the posterior cervical muscle spaces and fixing the opened middle space with self-developed titanium plates. The preliminary patient cohort showed that the posterior muscles were well preserved resulting in minimal axial symptoms as well as a good quality of life.

## Materials and methods

This study retrospectively analyzed 22 patients who underwent posterior modified double door laminoplasty at the Spine Surgery Department of Qilu Hospital of Shandong University from January 2018 to December 2020. Patients were included if they had cervical spondylotic myelopathy (CSM) caused by cervical disc herniation, cervical spinal stenosis and ossification of posterior longitudinal ligament. Patients with cervical spine fractures, cervical spinal cord injuries, spinal cord tumors, and patients who underwent anterior cervical spine fusion at the same time or earlier were excluded. All patients were followed up for at least 1 year, and CT images were reviewed at three and 12 months after surgery, whereas MRI images were reviewed at 12 months after surgery.

**Figure 1:**
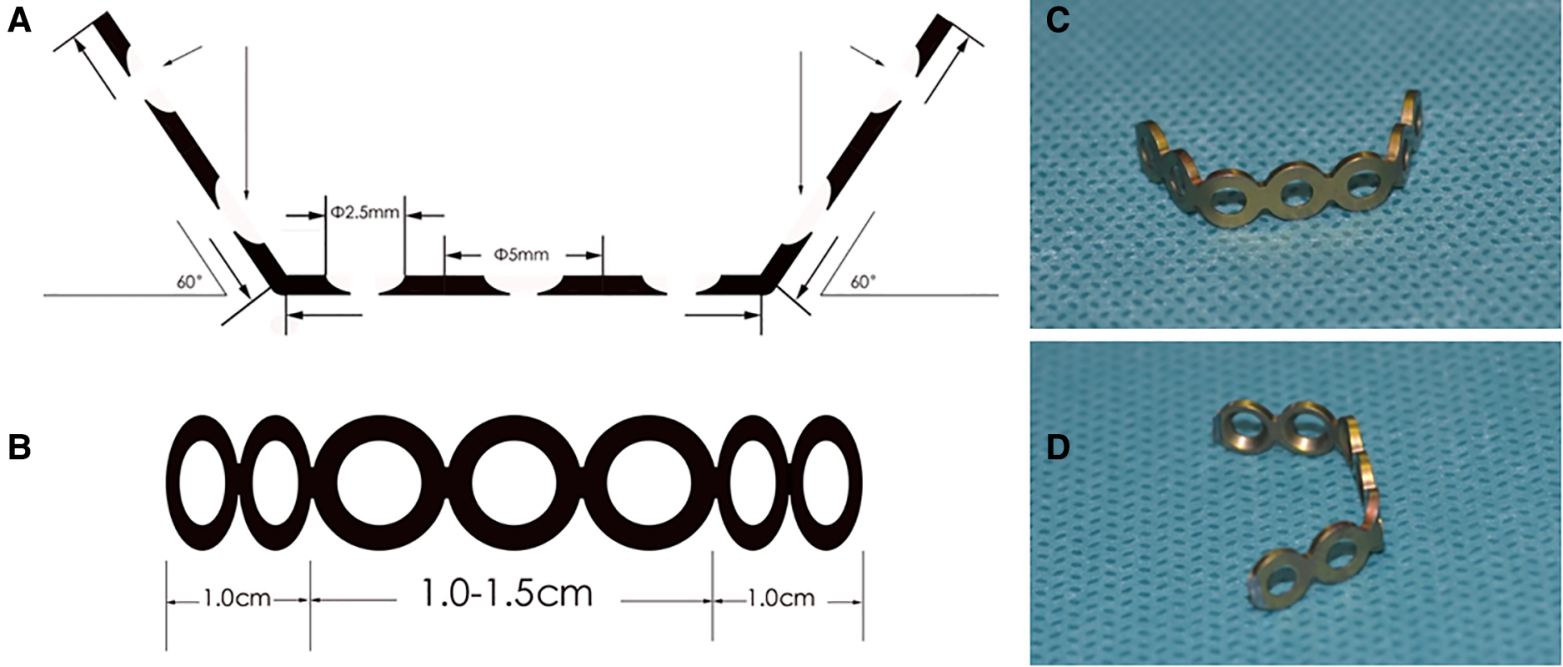
The schematic and photo images of self-developed mini titanium plate. (**A**) the transverse schematic image of mini plate showing the bending angle and the design of the nail holes; (**B**) the upper-view of the plate showing the length; (**C,D**) different views of the plate in photo.

**Figure 2:**
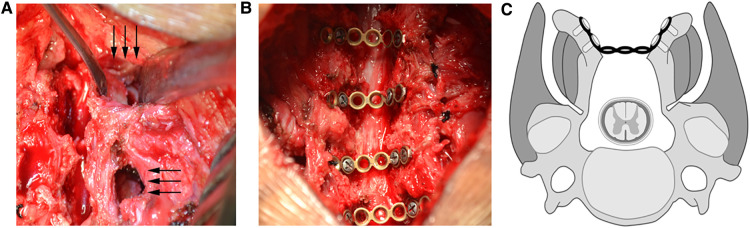
Schematic diagram of surgery and intraoperative photos. (**A**) a single segment of the unilateral lamina was separated from the cervical semispinalis muscle and the multifidus muscle space. Two mini retractors were used to maintain the operating window of the muscle space to create the laminar groove with extended high-speed drill; (**B**) the spinous process with semispinous muscles attached to them on both sides was spread and the bilateral titanium plate wings were fixed on the inner side of the spinous process to complete the fixation. (**C**) schematic diagram of surgery showing the creation of bilateral laminar grooves from muscle spaces, as well as the central fixation of separated spinous process.

The surgical procedure was modified on the Shirashi method. Under general anesthesia, the patient was placed in prone position, and a median incision was made at the back of the neck. The deep fascia was incised longitudinally. After median separation of the nuchal ligament, a single segment of the unilateral lamina was separated from the cervical semispinalis muscle and the multifidus muscle space. Two mini retractors were used to maintain the operating window of the muscle space. An extended high-speed drill was used to cut the laminar at the medial side of the lateral mass joint (transitional area). The outer cortex of the lamina was removed, retaining the inner plate to form a hinge structure. In the same way, similar grooves were created on both sides. Then, the spinous process bifurcation at the central division of the nuchal ligament was identified and using a drill the spinous process was split in the middle from the bifurcation. The spinous process with semispinous muscles attached to them on both sides was spread. The bilateral grooves were used to form a hinge for the door. A mini titanium plate with holes for screw-fixing that could adjust the width of the central spaces had been developed (Figure 1). Two different models of plate were prepared with two or three screw holes in the central part, making the length of central part as 1.0–1.5 cm. The length of plate could also be reduced by cutting off one screw hole on either or both ends. The screws were designed with diameter of 2.5 mm and various length of 5, 6, 7 and 8 mm. According to the width of the door, mini plate with two or three central holes was selected, and the bilateral titanium plate wings were fixed on the inner side of the spinous process to complete the fixation (Figure 2). For most circumstances, two screws were recommended on both wings. The ventral screw could be implanted with longer length as 7 mm or 8 mm. The dorsal screw was recommended with length of 5 or 6 mm. For C3 and C4 with small spinous process, the ventral screw of bilateral wings of the plate be implanted along the direction of lamina, providing enough strength for the effective fixation. With the effective strength of this screw, the dorsal screw is not essential in case of insufficient remaining spinous process.

All patients could sit up 48 h after the operations. Cervical collars were required for two weeks. The VAS scores of the patients were recorded postoperatively, two weeks, four weeks, 3 months and 12 months post operation. The JOA score was used to record the severity and recovery of spinal cord lesions in patients before, 3 months after and 12 months after surgery. The recovery was calculated by (Postoperative – preoperative score)/(17 – preoperative score)*100. The QoL scale (4) was used to evaluate the quality of life of the patients at 3 months and 12 months after surgery.

The x-ray examination was performed at 3 months and 12 months after operation, and the overall curvature and range of motion of C2–C7 in neutral position, hyperextension and hyperflexion position were recorded. CT scans and MRI were performed before, 3 months and 12 months after surgery. Changes in sagittal diameter of spinal canal before and after operation were measured by CT scans. MRI was used to measure the cross-sectional area of cervical paravertebral muscles before and after operation for assessment of the muscle atrophy.

Statistical analysis was performed using SPSS 22 statistical software. Paired T tests were used to test whether differences between corresponding continuous variables pre-op and post-operation. F test was used to test the homogeneity of variance. Difference were significant at a level of *p* < 0.05.

## Results

All 22 patients successfully underwent the operation. The mean operation time, blood loss and follow-up durations were 117 ± 25 min (SD), 149 ± 32 ml (SD) and 16.1 ± 3.6 months (SD) respectively. There were no complications such as dural sac tear nor nerve root injury during the operation. Surgical level of cervical spine mainly involved from C3-C7, while C2 was also involved in one patent. Two patients had incision exudate after surgery, which was cured after prolonged dressing change with no postoperative infection occurred. One patient developed C5 palsy and recovered after one month of neurotrophic treatment without revision surgery. All patients were immobilized for 48 h on the bed with muscle exercises. Drainage was removed 48 h post-surgery and the patients were encouraged to off-bed rehabilitation with cervical collar for two weeks. Then cervical spine mobility training was encouraged after four weeks post-surgery (Table 1).

**Table 1:** Patient demographics in this study.

Demographics	Patients Number = 22
Male/Female	10/12
Age (years)	61.6 ± 8.6 (52–78)
Operation time (min)	117 ± 25
Blood loss (ml)	149 ± 32
Follow-up period (months)	16.1 ± 3.6

There was no postoperative neurological deterioration in any patient. The preoperative, 3-month postoperative and 12-month postoperative JOA scores were 9.35 ± 3.25, 13.74 ± 4.86 and 15.73 ± 5.19 respectively. The 3-month and 12-month improvement rate RR were 57.4% and 83.4% respectively. The VAS score was used to evaluate the postoperative incision pain and axial pain. Mean VAS scores before, 3 months after and 12 months after surgery were 1.81 ± 0.79, 2.82 ± 1.56 and 2.18 ± 1.34 respectively. The QoL scale was used to evaluate the quality of life of the patients and was 62.25 ± 12.45 and 64.95 ± 14.50 at 3 months and 12 months after surgery respectively.

**Figure 3:**
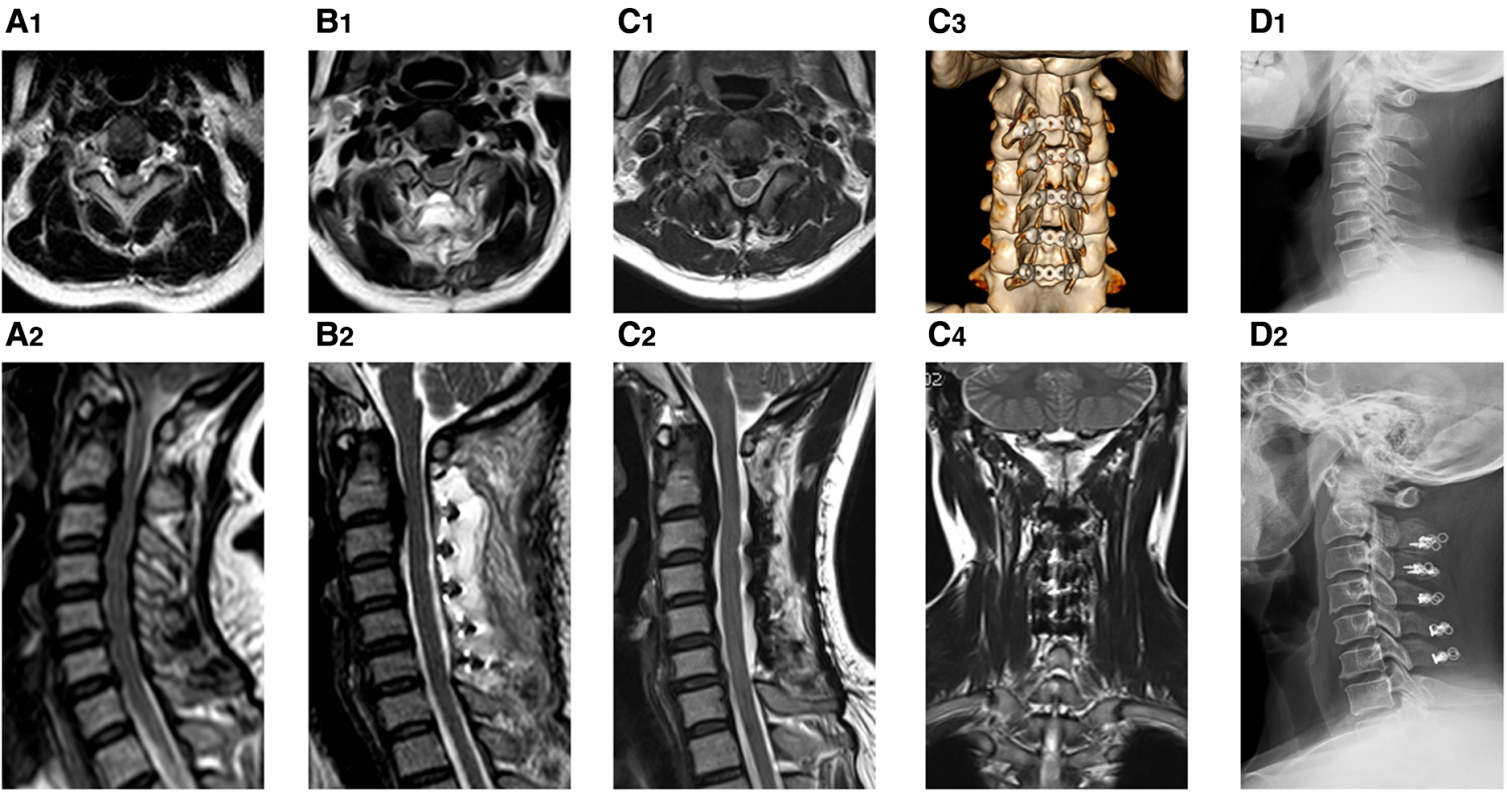
The pre-operative and post-operative images of the procedure. The patient was a 50-year-old female suffering CSM and OPLL. (**A1,A2**) the preoperative transverse and sagittal T2 images; (**B1,B2**) the postoperative images at 3 months post operation with transverse and sagittal T2 images; (**C1,C2**) the postoperative images at 12 months post operation with transverse and sagittal T2 images showing an obvious muscle recovery; (**C3,C4**) the 3D reconstruction CT images and coronal image of MRI at 12 months post operation; (**D1,D2**) the preoperative and postoperative standing lateral x-ray films at 12months follow-up.

The patients' cervical lordosis loss was assessed by x-ray. The C2–7 lordotic angle was 15.45 ± 9.15° and 13.75 ± 10.65° before and 12 months after surgery respectively. The C2–7 overall range of motion was 38.25 ± 14.35° and 32.15 ± 13.08° preoperatively and 12 months postoperatively respectively. The average sagittal diameter of the cervical spinal canal measured by CT before operation and 12 months after operation was 9.15 ± 1.55 and 14.25 ± 1.46 mm respectively, and this difference was statistically significant (*p* < 0.05). At three and 12 months after the operation, there was no change in positions of plates and screws. The average area of cervical paravertebral volume measured preoperatively and 3 months post operation was 409.73 ± 97.05 and 346.41 ± 83.38 mm^2^ (84% of pre-operative value) respectively, and this difference was statistically significant (*p* < 0.05). This average area measured was 381.07 ± 91.92 mm^2^ at 12 months post-surgery (Figure 3), which was 93% of the preoperative value with no significant difference (*p* > 0.05) (Table 2).

**Table 2:** The neurological and functional results of patients recorded during 12 months follow-up.

	Pre-operation	Post- operation 3 months	Post- operation 12 months
JOA score (improve rate)	9.35 ± 3.25	13.74 ± 4.86* (57.4%)	15.73 ± 5.19* (83.4%)
VAS score	1.81 ± 0.79	2.82 ± 1.56	2.18 ± 1.34
QoL scale	57.83 ± 16.07	62.25 ± 12.45	64.95 ± 14.50
C2–7 lordotic angle	15.45 ± 9.15°	13.57 ± 9.02°	13.75 ± 10.65°
The C2–7 overall range of motion	38.25 ± 14.35°	34.84 ± 12.46°	32.15 ± 13.08°
Sagittal diameter of canal (mm)	9.15 ± 1.47	13.34 ± 1.91*	13.29 ± 1.83*
Paravertebral muscle volume (mm^2^)	409.73 ± 97.05	346.41 ± 83.38*	381.07 ± 91.92

The cervical lordotic angle, range of motion and sagittal diameter of canal was evaluated by CT. The average area of cervical paravertebral muscle volume was measured on MRI images. *p < 0.05 with statistical difference compared to pre-operative value.

## Discussion

In recent years, numerous modifications have been made to classical posterior cervical double-door laminoplasty. New exposure methods to preserve the posterior muscles, the mini titanium plate and the advent of various new spacers, have made this operation less invasive (14). Here, we introduced a satisfactory double-door laminoplasty technique modification, in which bilateral grooves were created through muscle spaces and a bespoke titanium plate was used to fix the spinous process as spacer.

In Asia, posterior cervical laminoplasty, both single-door and double-door laminoplasty, is now commonly used by a large number of surgeons. Early applications required full dissection of the posterior cervical muscle from spinous process and removal of the spinous process, resulting in more obvious axial symptoms (15). Although re-suturing the muscles relieved axial symptoms, the re-sutured muscles tended to have necrosis due to ischemia. The modified exposure method for preserving the posterior cervical muscles was proposed by Shirashi in 2002 (9). The method initially focused on preserving five groups of muscles connecting the top of the C2 spinous processes which was later extended to cover other cervical spinous processes (C3–7) (6). The most important muscles are the semispinalis and multifidus, and the rotator. The semispinalis and multifidus muscles are longer and more vertical in the coronal position. They form a larger moment arm for the spinous process when exerting force, which plays an important role in completing the neck extension and stabilizing the cervical spine.

These muscle preserving effects were confirmed in in numerous studies (3, 4, 16, 17) studies. In Kotani's study (4), the mean VAS scores at final follow-up were 2.2 in muscle preserving group compared to 4.3 in conventional group. The deep muscle area on MRI was significantly higher in muscle preserving group than conventional group (102% vs. 58%). After that, several paper reported the adaptation of this improved exposure techniques in unilateral open-door laminoplasty, not double door laminoplasty. In Chen's study (18), the cervical curvature and ROM were significantly better in modified group compared to control group, while there were still decrease compared to pre-op values. The preservation of cervical muscle was significant in hinge side, with no difference in open side. This was due to the fact that during unilateral open-door laminectomy the laminar door was created in the longitudinal multi-segment continuous space on one side of the spinous process, while the hinge was created on the other side (10). However, the attachment of multifidus is still segmental, making complete preservation of the whole group of multifidus difficult. Double-door laminectomy avoids the excision of spinous process, which not only preserves the central attachment site of muscles but also completes the spinal cord decompression in the space created by splitting spinous process (16). A multi-segment continuous space is not required for preservation of the shape and function of muscles (17). The preservation of muscles could be bilateral in double-door laminoplasty, while mainly in hinge side in unilateral open-door laminoplasty. Therefore, we believe that Shirashi's method is especially suitable for double-door laminectomy. In our patients, postoperative MRI showed that the muscle shape and volume were maintained at good levels at 12 months, corroborating the muscle preserving possibility of double-door laminectomy. Indeed, there were still decrease in cervical lordosis and ROM after operation, indicating that this modified exposure technique could reduce the loss of function of muscles and tension band in the neck, not eliminate that. This was also comparable to the above previous studies.

The spacers in double-door surgery could maintain the width of the door and the sagittal diameter of the spinal canal after the door is opened. The autologous bone and hydroxyapatite spacers are a possible bridge for growth of the bilateral lamina (19). However, all spacers take time to fuse with the bilateral lamina, and have the risk of dissolution and rupture. Loosening of spacers may lead to displacement of the spacer, causing serious neurological complications (11). Therefore, mini titanium plates were recommended as substitutes for spacers in fixing bilateral lamina (13). Our self-designed mini titanium plate adjusted the length on both sides and central width of the plate during the operation, to adjust to the width of the central slot of each segment (Figure 1). The screws could immediately stabilize the lamina. At 12-month follow-up post-surgery, no patient had any displacement of their plates, corroborating titanium plates completely stabilize the lamina. This rigid stabilization improves the tension of the ligamentum flavum-lamina complex, thereby reducing incidences of postoperative axial pain (19). Few paper reported the experience of spacers in double-door laminoplasty with the modified muscle-preserving technique. Noguchi (19) reported use of hydroxyapatite spacers in double-door laminoplasty with traditional exposure technique. Guo (20) reported use of titanium plates in unilateral open-door laminoplasty with modified expose technique. As a supplement to these studies, it is our aim was to provide some experience with spacers in double-door laminoplasty using modified exposure techniques.

Currently, atrophy of the lumbar back after lumbar spine surgery, is a highly researched topic (21), while more attention is required in the muscled of cervical region. In this study, we compared cross-sectional MRI data of muscle groups of the neck before and after surgery, which confirmed the protective effect on the muscles. The preservation of muscle function after surgery is possibly dependent upon splitting of the spinous process, retaining the muscle insertion point, and completing the groove on both sides through the muscle spaces to reduce muscle dissection and ischemia. Moreover, after the lamina is firmly fixed with the titanium plate, the patient needs no cervical collar and this ultimately better maintains the patient's muscle function and shape (22). However, more research on concrete associations of muscle changes with axial pain in needed.

This study had limitations. First, due to the use of self-developed titanium plates, few patients were enrolled in this study for safety concern. Second, there was no comparative analysis between patients using titanium plates and patients using spacers to determine which was superior. Third, long-term follow-ups (2–5 years) are required observe the final survival of titanium plates in patients.

In this paper, we introduced a modified posterior cervical double-door laminoplasty based on Shirashi's method. The spinous process was split while retaining the muscle attach point, and laminar grooves were made on both sides through the segmental muscle space. After opening the door, the central gap was fixed with a self-developed titanium mini plate. Patients who underwent this surgical approach had preserved posterior muscles and this prevented obvious axial symptoms and improved their quality of life. Our findings provide a baseline for further research with larger cohorts.

## Data Availability

The original contributions presented in the study are included in the article/Supplementary Material, further inquiries can be directed to the corresponding author/s.
